# Highly specific ubiquitin-competing molecules effectively promote frataxin accumulation and partially rescue the aconitase defect in Friedreich ataxia cells

**DOI:** 10.1016/j.nbd.2014.12.011

**Published:** 2015-03

**Authors:** Alessandra Rufini, Francesca Cavallo, Ivano Condò, Silvia Fortuni, Gabriella De Martino, Ottaviano Incani, Almerinda Di Venere, Monica Benini, Damiano Sergio Massaro, Gaetano Arcuri, Dario Serio, Florence Malisan, Roberto Testi

**Affiliations:** a Laboratory of Signal Transduction, Department of Biomedicine and Prevention, University of Rome “Tor Vergata,” Via Montpellier 1, Rome 00133, Italy; b Fratagene Therapeutics Ltd., 22 Northumberland Rd., Dublin, Ireland; c Department of Experimental Medicine and Surgery, University of Rome “Tor Vergata,” Via Montpellier 1, Rome 00133, Italy

**Keywords:** Friedreich ataxia, Frataxin, Ubiquitin, Orphan drug development

## Abstract

Friedreich ataxia is an inherited neurodegenerative disease that leads to progressive disability. There is currently no effective treatment and patients die prematurely. The underlying genetic defect leads to reduced expression of the mitochondrial protein frataxin. Frataxin insufficiency causes mitochondrial dysfunction and ultimately cell death, particularly in peripheral sensory ganglia. There is an inverse correlation between the amount of residual frataxin and the severity of disease progression; therefore, therapeutic approaches aiming at increasing frataxin levels are expected to improve patients' conditions. We previously discovered that a significant amount of frataxin precursor is degraded by the ubiquitin/proteasome system before its functional mitochondrial maturation. We also provided evidence for the therapeutic potential of small molecules that increase frataxin levels by docking on the frataxin ubiquitination site, thus preventing frataxin ubiquitination and degradation. We called these compounds ubiquitin-competing molecules (UCM). By extending our search for effective UCM, we identified a set of new and more potent compounds that more efficiently promote frataxin accumulation. Here we show that these compounds directly interact with frataxin and prevent its ubiquitination. Interestingly, these UCM are not effective on the ubiquitin-resistant frataxin mutant, indicating their specific action on preventing frataxin ubiquitination. Most importantly, these compounds are able to promote frataxin accumulation and aconitase rescue in cells derived from patients, strongly supporting their therapeutic potential.

## Highlights

• New therapeutic strategy to treat Friedreich ataxia
• New ubiquitin-competing molecules (UCM) directly target frataxin protein
• UCM prevent frataxin ubiquitination and degradation
• UCM increase frataxin and rescue aconitase in cells derived from patients

## Introduction

Friedreich ataxia (FRDA) is a genetic neurodegenerative disease that affects children and young adults and leads to progressive disability and premature death (Pandolfo and Pastore, 2009). It has an autosomal recessive inheritance with an estimated prevalence of 1:50,000 in the Caucasian population, thus representing the most common form of inherited ataxia. Characteristic symptoms include progressive loss of movement coordination, gait instability, muscle weakness and sensory loss. Symptoms usually appear around puberty and patients usually require wheelchairs within 10 to 15 years from disease onset. Disease progression is often associated with loss of visual acuity, slurred speech and dysphagia. Neurological signs are associated with degeneration of sensory neurons in the dorsal root ganglia and dentate nucleus of the cerebellum. Moreover, patients often develop a hypertrophic cardiomyopathy that is often the cause of premature death (Weidemann et al., 2013). A significantly higher incidence of diabetes mellitus is also associated with the disease, with more than 25% of patients developing glucose intolerance or diabetes (Cnop et al., 2013). The disease is caused by a GAA triplet-repeat expansion within the first intron of the gene coding for frataxin (Campuzano et al., 1996), which results in reduced transcription of the gene. The vast majority of patients present a homozygous repeat expansion on both alleles, while about 4% of patients present a GAA repeat expansion on one allele and a point mutation in the coding region on the other allele. In normal individuals the number of GAA triplets range between 10 and 35, while in affected individuals GAA triplets range from 66 up to more than 1700 (Yandim et al., 2013). This is associated with a number of epigenetic changes that lead to heterochromatinization of this portion of DNA and impairment of gene transcription (Al-Mahdawi et al., 2008; Greene et al., 2007). The formation of an atypical “sticky” triple DNA structure has also been found associated with the expanded GAA triplet, also accounting for the observed gene silencing (Bidichandani et al., 1998; Sakamoto et al., 2001). The genetic defect results in a severely reduced transcription of the frataxin gene, with patients living with residual 10-30% frataxin (Yandim et al., 2013).

Frataxin is a highly conserved mitochondrial protein (Pastore and Puccio, 2013), synthesized in the cytosol as a precursor form, which is then processed by a mitochondrial processing peptidase in a two-step catalytic process, and concomitantly imported into mitochondria (Koutnikova et al., 1998). The functional mature form is a 130 amino acid polypeptide (Condò et al., 2007), mainly located within the mitochondrial matrix. Frataxin is involved in iron metabolism and participates in the biogenesis of iron-sulfur clusters (ISC) (Vaubel and Isaya, 2013). It is therefore essential for the enzymatic activity of complex I, II and III of the mitochondrial respiratory chain and of aconitases (Bulteau et al., 2004; Condò et al., 2010; Rotig et al., 1997), which require ISC as cofactors. Therefore, frataxin-deficient cells present an impairment in the electron transport chain and inefficient mitochondrial respiration. Iron distribution is consequently affected in frataxin-deficient cells, resulting in intramitochondrial iron overload (Martelli and Puccio, 2014). As a result of dysregulated mitochondrial metabolism, frataxin-deficient cells have reduced ATP content and increased ROS generation. Moreover, frataxin insufficiency results in impaired intracellular anti-oxidant defenses (Paupe et al., 2009; Shan et al., 2013).

To date, no effective therapy has been approved to treat FRDA. Frataxin insufficiency is considered the main pathogenic cause of the disease and a correlation exists between the length of the GAA expansion and the extent of transcription impairment. Moreover, the amount of residual frataxin has been correlated to the age at disease onset and to the severity of disease progression (Parkinson et al., 2013). Since the coding sequence of frataxin is unaltered in patients, therapeutic approaches aiming at increasing frataxin levels (Herman et al., 2006; Marmolino et al., 2009; Tomassini et al., 2012) are therefore expected to ameliorate disease symptoms and to slow down disease progression. Both promoting frataxin gene expression and preventing frataxin protein degradation can in principle lead to an increase in frataxin levels. We recently focused our studies on the therapeutic potential of preventing frataxin degradation to promote its accumulation.

We have previously shown that frataxin levels are controlled by the ubiquitin/proteasome system (UPS) and that frataxin can be directly modified by ubiquitin (Rufini et al., 2011). The UPS is the most important and widely studied system for intracellular protein degradation. Lysine residues on the protein substrate are recognized by specific enzymes and modified by the covalent attachment of one or more ubiquitin moieties. This event marks the protein for degradation in the proteasome (Glickman and Ciechanover, 2002). We identified the critical lysine on frataxin, lysine 147 (K147), which is the main target of ubiquitination. This lysine represents a crucial site for frataxin stability. Indeed a frataxin mutant that lacks this lysine cannot be ubiquitinated and is more stable. Therefore, preventing ubiquitination on K147 is expected to grant frataxin an increased stability and a prolonged half-life. Indeed, from computational docking studies, we identified a set of small molecules, predicted to interact with the molecular pocket surrounding K147, able to interfere with frataxin ubiquitination and promote frataxin accumulation in cells derived from patients. Moreover, these molecules, named ubiquitin-competing molecules (UCM), can promote a functional rescue of mitochondrial dysfunction caused by frataxin deficiency in patients cells (Rufini et al., 2011).

We have now extended our search to find more effective compounds. We show here that these “second-generation” UCM, physically interact with frataxin and prevent its ubiquitination. Moreover, these compounds can increase frataxin levels in cells overexpressing frataxin, but not in cells overexpressing the ubiquitin-resistant K147R frataxin mutant, suggesting that they act by inhibiting ubiquitination on K147. Importantly, they show efficacy in promoting accumulation of mature frataxin, and in restoring aconitase activity in cells derived from patients, strongly supporting their potential therapeutic application.

## Results

### Computational screening for ubiquitin-competing molecules (UCM)

**Fig. 1:**
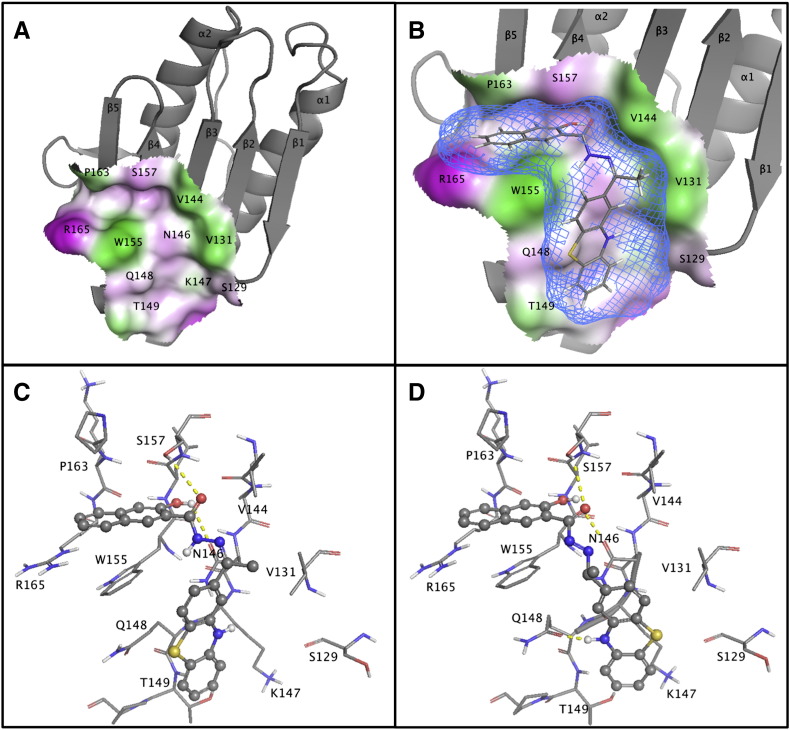
Docking model of UCM71 to frataxin. (A) Crystal structure of frataxin (gray cartoon) with solvent accessible molecular surface around the W155 pocket. Overall structure as grey cartoon, molecular surface colored by lipophilicity (hydrophilic in magenta, lipophilic in green). (B) Putative model of the interaction between W155 pocket and the ligand UCM71 (ball and stick, CPK colors): the solvent accessible ligand surface (light blue mesh) fits perfectly with the naphthyl moiety buried by W155, P163, and S157. The phenothiazine ring recognizes the flat region formed by N146 and K147 (label not shown for clarity) and delimited by side chains of V131, S129, T149 and Q148. (C) Putative selected interactions of UCM71 with frataxin. Hydrogen bonds are formed between the hydroxyl substituent of the naphthyl group and the side chain of N146 and between the carbonyl group of the carbonyl-hydrazone scaffold and the hydroxyl of S157. These interactions induce minor rearrangements in the involved side chains of N146 (flip of terminal amide) and S157. (D) The flexibility of the carbonyl-hydrazone scaffold permits the flip of phenothiazine moiety to point its central amino group toward Q148 possibly inducing the flip of the amide group of its side chain resulting in the formation of a strong hydrogen bond.

In order to design small molecules able to inhibit the ubiquitination of frataxin, an extended analysis of the protein's accessible surfaces has been performed, extending our previous work, by taking into account protein flexibility. This analysis allowed us to identify binding pockets on frataxin that were more accessible to drugs. By focusing our analysis on the areas more proximal to K147, and by using virtual screening of commercially available compound libraries, several thousand compounds were docked on available NMR and x-ray structures of human frataxin. Some of these molecules were predicted to interact with frataxin near to K147 (Fig. 1). Promising candidates were subjected to functional validation.

### UCM increase frataxin levels

**Fig. 2:**
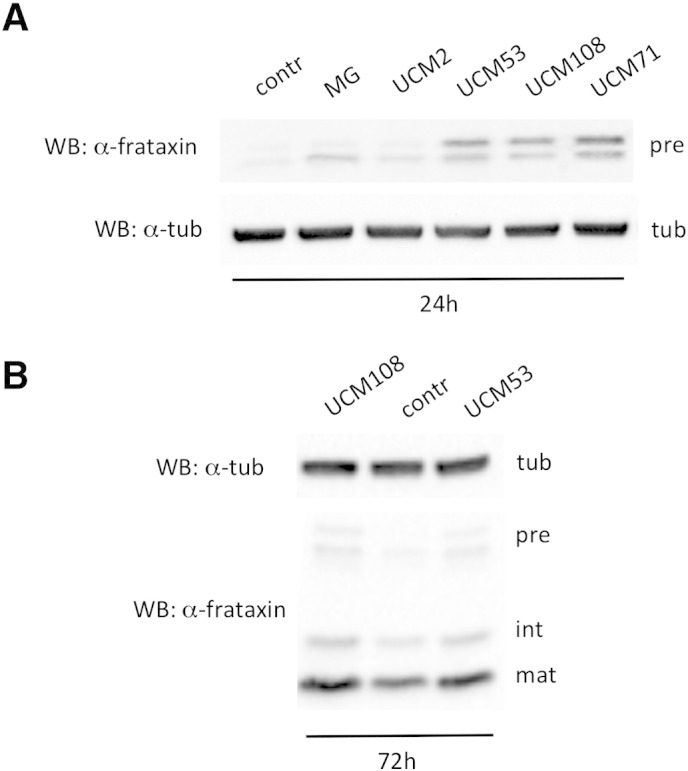
UCM increase frataxin levels. (A) To detect frataxin precursor accumulation, 293 Flp-In cells stably expressing frataxin^1 -210^ were treated for 24 hrs with 10 μM of the indicated UCM or 10 μM MG132 (MG). Total cell extracts were resolved on SDS–PAGE and analyzed with anti-frataxin antibody, or anti-tubulin, as a loading control. Pre: precursor; tub: tubulin. (B) To detect mature frataxin accumulation, 293 Flp-In cells stably expressing frataxin^1 -210^ were treated for 72 hrs with 10 μM of the indicated UCM. Total cell extracts were resolved on SDS–PAGE and analyzed with anti-frataxin antibody, or anti-tubulin, as a loading control. Pre: precursor, int: intermediate, mat: mature; tub: tubulin.

To validate UCM activity, we tested their effect in human HEK-293 cells stably expressing single copy frataxin (293-frataxin). These cells allow the detection of all forms of frataxin, including the frataxin precursor. Compounds that were able to enhance frataxin precursor levels were further chemically modified to better fit the docking model, synthesized and tested again in 293-frataxin. This process was repeated in an iterative cycle with the aim to improve the efficacy of the UCM. Approximately 200 new candidate UCM were tested in functional assays. Through this process, we were able to identify new UCM that promote frataxin precursor accumulation more efficiently than the previously described compounds. Structures of the compounds described in this study are shown in Table 1. Indeed, the treatment of 293-frataxin cells with 10 μM UCM53, UCM108 and UCM71 is able to induce frataxin precursor accumulation (Fig. 2A) more efficiently than with the previously described UCM2 (referred to as NSC620301 in (Rufini et al., 2011)) or with the proteasome inihibitor MG132. Importantly, an accumulation of mature frataxin can also be observed in these cells when treatment is prolonged for 3 days (Fig. 2B).

### UCM prevent frataxin ubiquitination

**Fig. 3:**
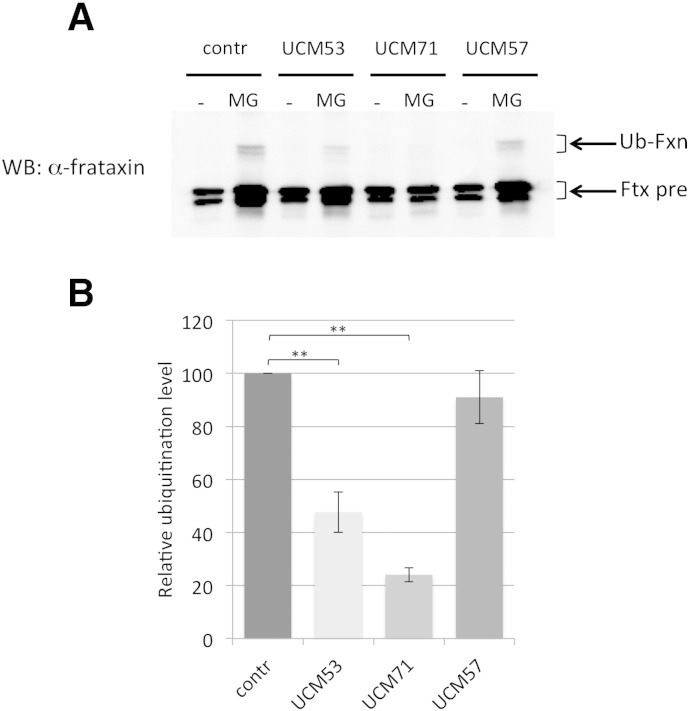
UCM prevent frataxin ubiquitination. (A) 293 cells were transiently co-transfected with HA-Ub and frataxin^1 -210^. Twenty-four hours after transfection, cells were treated with 10 μM of the indicated UCM or with DMSO alone (contr). UCM57 was used as a non-effective control molecule. Cells were harvested 48 h after transfection. Where indicated (MG), cells were also treated with 10 μM MG132 and 50 ng/ml ubiquitin-aldehyde for the last 16 h. Total cell extracts were resolved on SDS–PAGE and revealed with anti-frataxin antibody. The arrows indicate the bands corresponding to frataxin precursor (Fxn-pre) and ubiquitin-conjugated frataxin (Ub-Fxn). (B) The graph represents the relative ubiquitination levels, quantified as the densitometric ratio between ubiquitinated frataxin bands and frataxin precursor bands for each MG132-treated lanes. Data represent the mean ± SEM from five different independent experiments. *P-*values were calculated with Student's *t*-test and were statistically significant (**P < 0.01) compared to non-treated control.

To test whether the new compounds promote frataxin accumulation by preventing its UPS-dependent degradation, we evaluated their impact on frataxin ubiquitination. To this aim, we performed an *in vivo* ubiquitination assay. HEK-293 cells were transiently co-transfected with hemagglutinin-tagged ubiquitin (HA-Ub) and frataxin, in the presence of proteasome inhibitor and deubiquitinase inhibitor to allow the accumulation of ubiquitinated species, in the presence of the selected compounds. The ubiquitination status of frataxin was evaluated by SDS–PAGE of total cell lysates and anti-frataxin immunoblotting. As previously described, in this experimental setting, frataxin monoubiquitinated forms can be detected by anti-frataxin antibody as a slower migrating band above frataxin precursor (Rufini et al., 2011). Ubiquitination level was measured as the ratio between the levels of ubiquitinated frataxin and frataxin precursor. As shown in Fig. 3, UCM53 and UCM71 but not the control non-effective molecule UCM57, can significantly abrogate frataxin ubiquitination. These data suggest that the selected UCM interfere with frataxin ubiquitination in living cells.

### UCM promote frataxin accumulation by preventing K147-dependent degradation

**Fig. 4:**
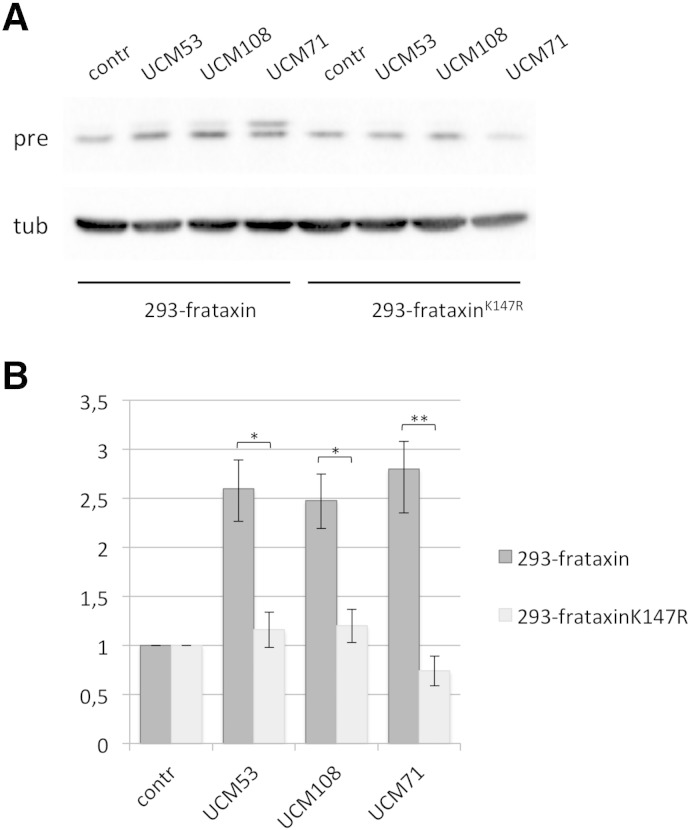
UCM promote frataxin precursor accumulation by preventing K147-dependent degradation. (A) 293 Flp-In cells stably expressing frataxin^1 -210^ (293-frataxin) or the lysine-mutant frataxin^K147R^ (293-frataxin^K147R^) were treated for 24 hrs with 10 μM of the indicated UCM. Proteins were resolved on SDS–PAGE and revealed with anti-frataxin antibody or anti-tubulin, as a loading control. Pre: precursor; tub: tubulin. (B) The graph represents relative frataxin precursor levels as quantified by densitometric analysis. Data represent the mean ± SEM from five different independent experiments. *P-*values were calculated with Student's *t*-test and were statistically significant (*P < 0.05; **P < 0.01).

We had previously shown that K147 is the crucial ubiquitination site on frataxin. Since we showed that these compounds are able to efficiently abrogate frataxin ubiquitination, we could anticipate that they act by interfering with ubiquitination on K147. Thus, to validate this hypothesis, we evaluated their effect on the frataxin mutant that lacks K147 (K147R). This mutant cannot be ubiquitinated and is therefore resistant to UPS-mediated degradation. Small molecules that act by preventing ubiquitination on K147 are expected to be ineffective on this mutant. We therefore tested their effect on HEK-293 cells stably expressing the ubiquitin-refractory frataxin^K147R^ mutant (293-frataxin^K147R^). Cells were treated for 24 h with the indicated compounds and frataxin precursor levels analyzed by western blot on total cell extract. Indeed, we could show that when 293-frataxin^K147R^ are treated with the selected UCM, no significant increase in frataxin precursor levels can be detected (Fig. 4), compared to what observed in 293-frataxin, expressing wild-type frataxin. These data suggest that UCM act on frataxin by interfering with the K147-dependent degradation pathway.

### UCM interact with frataxin

**Fig. 5:**
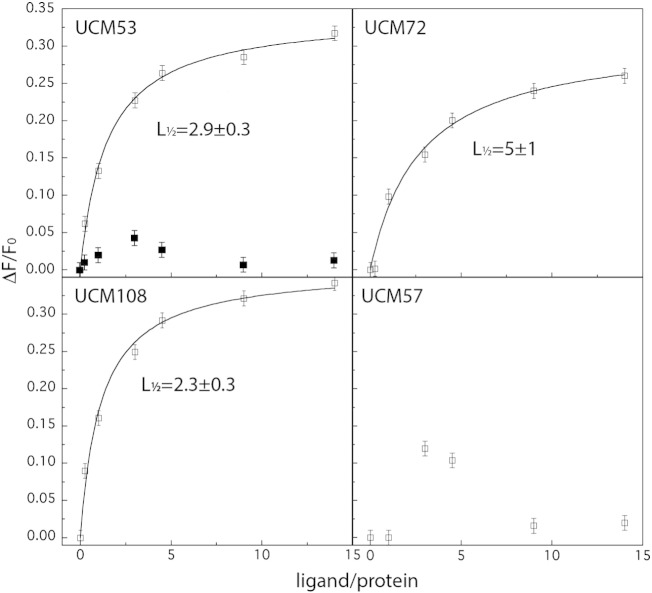
UCM interact with frataxin. Fluorescence studies of the interaction of 2 μM recombinant frataxin precursor with different concentration of the indicated UCM (ligand). The graphs represent the fractional loss of the protein fluorescence intensity (ΔF/F_0_), in the presence of different concentration of ligand, *versus* the ratio between ligand and frataxin. The interaction with UCM53 was also analyzed in the case of frataxin precursor previously denaturated in 3 M guanidinium hydrochloride (upper left panel, black square symbols). The half-saturation binding constant, L_1/2_ (μM), of frataxin precursor with UCM53, UCM108 and UCM72 is indicated in each corresponding panel.

Ubiquitin-competing molecules were selected through structure-based virtual screening for their potential ability to interact with frataxin on its ubiquitination site. Since they are in fact able to prevent frataxin ubiquitination and to interfere with its K147-dependent degradation, we wanted to confirm their ability to physically interact with frataxin. Therefore, the interaction propensities of frataxin with these molecules were investigated by fluorescence spectroscopy through the analysis of the changes of the signal of the protein tryptophan residues in the presence of the different compounds. In Fig. 5, we have reported the binding isotherm of the protein to four different compounds, UCM53 and UCM108 that promote frataxin accumulation, and UCM72 and UCM57 that are unable to promote frataxin accumulation (see below), as a control. One of the effective compounds (UCM53) was also exposed to the denatured protein. The half-saturation binding constant, L_1/2_, for UCM53, UCM108 and UCM72 are reported in each corresponding panel. UCM53 and UCM108 showed strong fluorescence changes (left panels) and the lowest binding constants, while the control UCM72 showed the highest binding constant (upper right panel). Fluorescent changes induced by UCM53 were completely lost using the denatured protein (upper left panel, black square symbols). The interaction of frataxin with the control molecule UMC57 was also studied by fluorescence (lower right panel). In this case, the spectroscopic properties of the protein do not significantly change in the presence of this compound obtaining similar results to those of denatured protein + UCM53 (upper left panel, black squares). These results indicate that UCM53 and UCM108 strongly interact with tryptophans likely due to a quite efficient binding.

### UCM promote frataxin accumulation in FRDA cells and rescue the aconitase defect

**Fig. S1:**
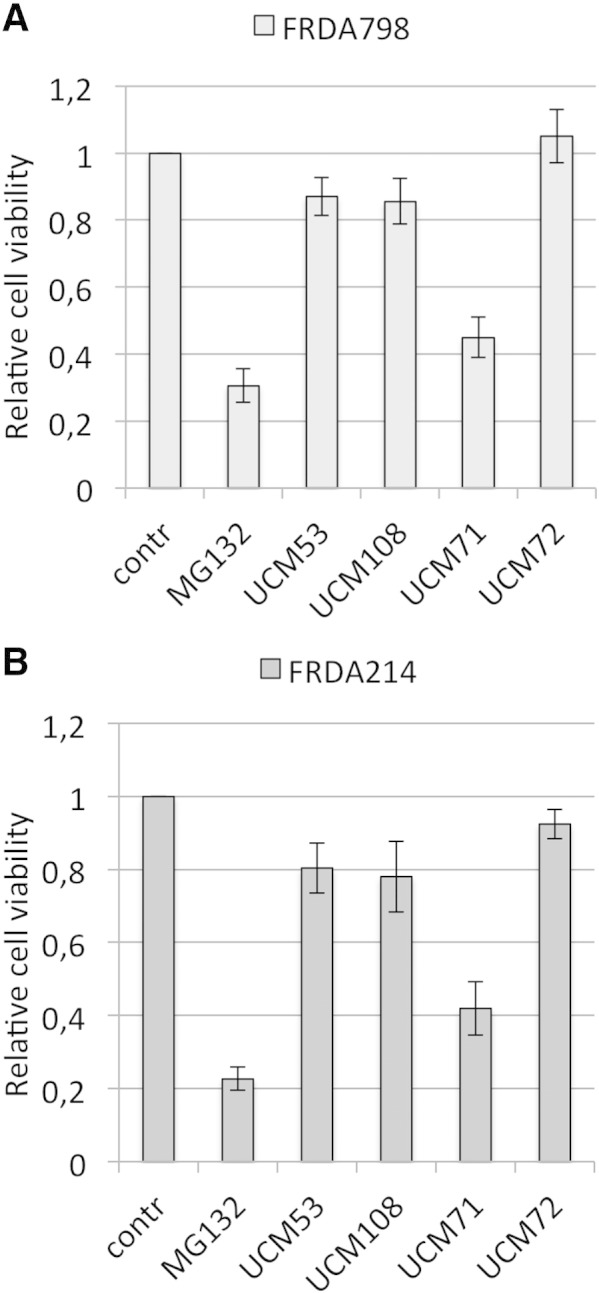
Effect of UCM on cell viability. FRDA patients-derived lymphoblasts cell lines FRDA 798 **(A)** and FRDA 214 **(B)** were cultured in the presence of 10 μM of the indicated UCM, MG132 or DMSO alone (contr) for 5 days. Toxicity of the indicated compounds was measured with the XTT assay (Sigma-Aldrich) and expressed as relative cell viability compared to control untreated cells.

**Fig. S2:**
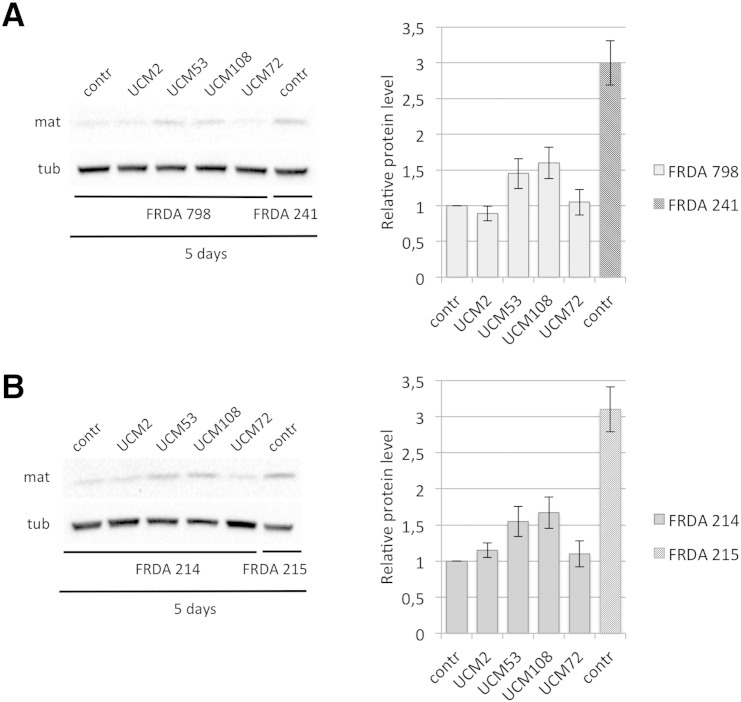
UCM53 and UCM108 are more effective than the previously described compound. FRDA patients-derived lymphoblasts cell lines FRDA 798 **(A)** and FRDA 214 (B) or lymphoblasts derived from the corresponding unaffected carrier siblings, FRDA 241 or FRDA 215 respectively, were cultured in the presence of 10 μM of the indicated UCM, or DMSO alone (contr) for 5 days. Total cell extracts were resolved on SDS–PAGE and analyzed with anti-frataxin antibody, or anti-tubulin, as a loading control. The graphs represent the relative frataxin abundance as quantified by densitometric analysis and normalized with tubulin levels. Data represent the mean ± SEM from three different independent experiments. Tub: tubulin; mat: mature frataxin.

**Fig. 6:**
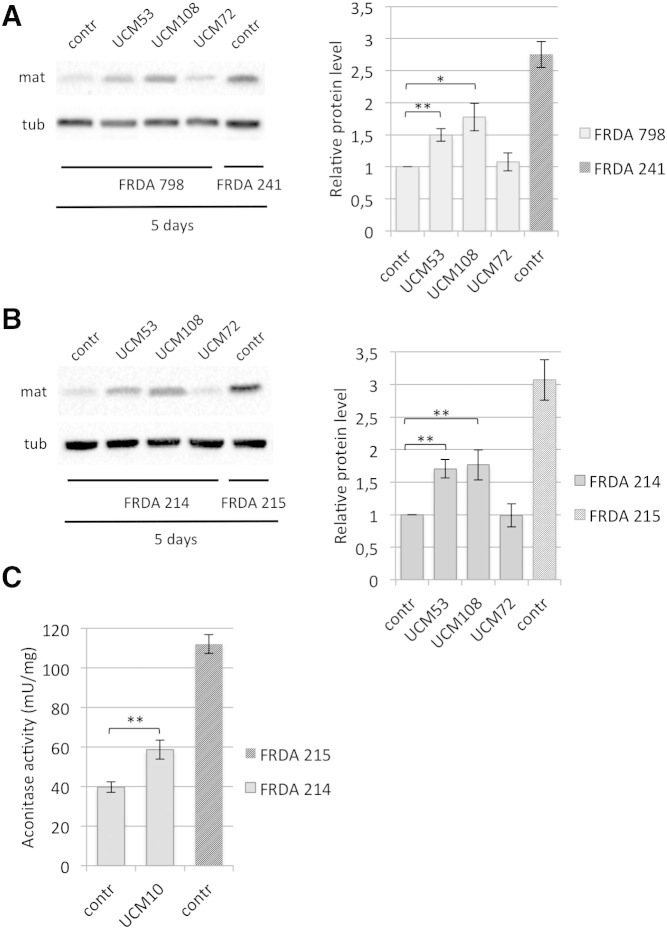
UCM promote frataxin accumulation in FRDA cells and rescue the aconitase defect. FRDA patients-derived lymphoblasts cell lines FRDA 798 (A) and FRDA 214 (B) or lymphoblasts derived from the corresponding unaffected carrier siblings, FRDA 241 or FRDA 215, respectively, were cultured in the presence of 10 μM of the indicated UCM, or DMSO alone (contr) for 5 days. Total cell extracts were resolved on SDS–PAGE and analyzed with anti-frataxin antibody, or anti-tubulin, as a loading control. The graphs represent the relative frataxin abundance as quantified by densitometric analysis and normalized with tubulin levels. Data represent the mean ± SEM from four different independent experiments. *P-*values were calculated with Student's *t*-test and were statistically significant (*P < 0.05; **P < 0.01) compared to non-treated conditions. Tub: tubulin; mat: mature frataxin. (C) Patients-derived lymphoblasts FRDA 214 were cultured with DMSO alone (contr) or in the presence of 10 μM of UCM108 for 5 days. The unaffected carrier siblings FRDA 215 lymphoblasts were cultured with DMSO alone. Total aconitase activities were measured and normalized as described in the Methods section. Data represent the mean ± SEM from four different independent experiments. *P-*values was calculated with Student's *t*-test and was statistically significant (**P < 0.01) compared to non-treated condition.

We finally analyzed the efficacy of our compounds in cells derived from FRDA patients. Lymphoblast cell lines derived from two different patients, FRDA 798 and FRDA 214 were cultured for 5 days in the presence of 10 μM of UCM53 or UCM108, the two most promising compounds. UCM71 could not be evaluated because of its toxicity over a long-term treatment (Fig. S1). Frataxin levels were quantified by western blot analysis on whole cell extracts. Importantly, we could observe a significant accumulation of mature frataxin when cells are cultured in the presence of UCM53 or UCM108, compared to cells treated with vehicle alone (control), or UCM72 (Figs. 6A and B). Frataxin levels in treated cells derived from patients are also compared to the levels observed in cells derived from unaffected carrier siblings (FRDA 241 and FRDA 215). These UCM are also more effective than the previously described compounds (Rufini et al., 2011). Indeed, at the concentration of 10 μM that is efficacious for the new UCM, the previously described compound does not show a significant effect (Fig. S2). Moreover, to validate a functional recovery of frataxin levels, rescue of cellular aconitases activity was evaluated in FRDA cells upon treatment with UCM. A significant increase in aconitases activity was observed in patient-derived lymphoblast cell line FRDA 214 after treatment with UCM108 for 5 days (Fig. 6C). Aconitases activity in cells derived from an unaffected carrier sibling (FRDA 215) is also shown for comparison. Thus, importantly, these data indicate that treatment with UCM allows accumulation of a functional form of mature frataxin with consequent reactivation of ISC biogenesis.

We finally analyzed the efficacy of our compounds in cells derived from FRDA patients. Lymphoblast cell lines derived from two different patients, FRDA 798 and FRDA 214 were cultured for 5 days in the presence of 10 μM of UCM53 or UCM108, the two most promising compounds. UCM71 could not be evaluated because of its toxicity over a long-term treatment (Fig. S1). Frataxin levels were quantified by western blot analysis on whole cell extracts. Importantly, we could observe a significant accumulation of mature frataxin when cells are cultured in the presence of UCM53 or UCM108, compared to cells treated with vehicle alone (control), or UCM72 (Figs. 6A and B). Frataxin levels in treated cells derived from patients are also compared to the levels observed in cells derived from unaffected carrier siblings (FRDA 241 and FRDA 215). These UCM are also more effective than the previously described compounds (Rufini et al., 2011). Indeed, at the concentration of 10 μM that is efficacious for the new UCM, the previously described compound does not show a significant effect (Fig. S2). Moreover, to validate a functional recovery of frataxin levels, rescue of cellular aconitases activity was evaluated in FRDA cells upon treatment with UCM. A significant increase in aconitases activity was observed in patient-derived lymphoblast cell line FRDA 214 after treatment with UCM108 for 5 days (Fig. 6C). Aconitases activity in cells derived from an unaffected carrier sibling (FRDA 215) is also shown for comparison. Thus, importantly, these data indicate that treatment with UCM allows accumulation of a functional form of mature frataxin with consequent reactivation of ISC biogenesis.

## Discussion

The present study supports the potential role of small molecules that prevent frataxin ubiquitin-dependent degradation as a novel therapeutic strategy to treat FRDA. There is currently no approved therapy for FRDA and only limited treatment for the management of symptoms are available for patients (Delatycki, 2009; Schulz et al., 2009). Since patients lack a sufficient amount of mitochondrial frataxin, increasing the amount of frataxin is the main therapeutic goal (Perdomini et al., 2014). We had previously shown (Rufini et al., 2011) that a significant portion of the frataxin precursor is degraded by the UPS before it reaches the mitochondria, where it undergoes maturation and becomes functional. We therefore reasoned that preventing frataxin precursor degradation could lead to an increase in the pool of frataxin available for mitochondrial import. Importantly, we had previously provided evidence for the therapeutic potential of small molecules that prevent frataxin ubiquitination by docking to the frataxin ubiquitination site (Rufini et al., 2011). We now extended our search to identify more effective compounds showing increased affinity toward frataxin and improved efficacy in cells derived from patients. Moreover, we provide important insight into the mechanism of action of these compounds. Indeed, the fact that these molecules are not effective in cells expressing frataxin^K147R^, a frataxin mutant lacking the critical ubiquitination site, strongly supports their predicted role in preventing ubiquitination on that particular lysine residue. In line with these data, we provide for the first time evidence for the direct binding of these compounds with frataxin. Indeed, fluorescence spectroscopy experiments indicate that selected compounds can physically interact with frataxin *in vitro*, as predicted by the *in silico* studies. Importantly, we could show that the activity of aconitase that strongly depends on frataxin is partially rescued upon treatment with UCM in cells derived from patients. These data suggest that interaction with UCM does not alter frataxin function. One of the compounds described in this paper, UCM71, however shows some toxic effect on cell viability and was therefore not considered for further studies. Further insight into the binding mode of these UCM to frataxin will be helpful for the future development of the identified leads and will guide the design of new derivatives with improved efficacy.

The function of the UPS is critical for the regulation of protein turnover and the maintenance of protein homeostasis. The stability of many intracellular targets is known to be altered in a number of diseases, including cancer and neurodegenerative disorders. Given the importance of the UPS in controlling protein stability and degradation, many components of the system represent important therapeutic targets (Bedford et al., 2011; Shen et al., 2013; Zhang and Sidhu, 2014). Several small molecule inhibitors that target different steps of the ubiquitination and degradation pathway have been developed. Among those, two proteasome inhibitors, Bortezomib and Carfilzomib, are now approved for cancer treatment (Kisselev et al., 2012; Rentsch et al., 2013; Richardson et al., 2006). However, targeting the proteasome as a whole is too unspecific and may result in many undesired outcomes. The identification of new and more specific therapeutic targets among UPS components is crucial for the design of more selective therapies. E3 ligases are the enzymes responsible for substrate recognition and confer specificity to the ubiquitination process. They therefore represent attractive and more selective pharmacological targets. A widely studied example in the field is the ubiquitination of p53 by its E3 ligase HDM2 (Kussie et al., 1996). Different strategies have been adopted to prevent p53 ubiquitination. A number of small molecule inhibitors have been developed that prevent p53/HDM2 interaction, either by binding to the p53-binding pocket on HDM2, like Nutlin3 (Vassilev et al., 2004) or MI-63 (Ding et al., 2006), or by inducing a conformational change on p53, like RITA (Issaeva et al., 2004). Alternatively, other compounds, like HLI98, directly bind to the HDM2 catalytic pocket, thus inhibiting its enzymatic activity (Roxburgh et al., 2012; Yang et al., 2005). Other small molecule inhibitors have been developed that target the activity of multi-subunit E3 ligase complexes, like the Cullin family of RING E3 ligases, which are responsible for the ubiquitination of a large number of intracellular substrates. MLN4924 prevents the neddylation of Cullins, a step required for their activation, thereby preventing the degradation of all Cullin substrates (Brownell et al., 2010; Soucy et al., 2009). Other compounds interfere with the assembly of the multi-subunit complex (Chen et al., 2008) or directly prevent interaction of the adaptor subunit with the substrate (Wu et al., 2012). However, to our knowledge, our therapeutic strategy represents the first example of a small molecule inhibitor developed to directly target ubiquitination site on the substrate protein to prevent its degradation. This approach could potentially be applied to prevent ubiquitination of other crucial intracellular substrates. As an alternative and complementary approach to prevent frataxin degradation, we are now pursuing the identification of the frataxin-specific E3 ligase, which will represent another attractive therapeutic target to treat FRDA.

## Methods

### Cell culture and transfections

Human embryonic kidney HEK-293 cells were cultured in Dulbecco's modified Eagle's medium (DMEM) supplemented with 10% fetal bovine serum (FBS). HEK-293 cells were transfected using Lipofectamine 2000 reagents (Invitrogen), according to the manufacturer's instructions. Cells were plated on 6 cm dishes and transfected with 8 μg of total DNA (4 μg of pIRES-frataxin and 4 μg of HA-Ub). The day after transfection, cells were treated for 24 h with 10 μM UCM together with 10 μM proteasome inhibitor MG132 and 50 ng/ml deubiquitinating enzyme (DUB) inhibitor Ub-aldehyde. HEK-293 Flp-In cells (Invitrogen) are HEK-293 variants allowing the stable and isogenic integration and expression of a transfected gene. The HEK-293 clones stably expressing frataxin^1–210^ or frataxin^K147R^ were previously described (Condò et al., 2007; Rufini et al., 2011). FRDA 214 (GM16214) and FRDA 798 (GM16798) lymphoblasts, from clinically affected FRDA patients, as well as FRDA 215 (GM16215) and FRDA 241 (GM16241) lymphoblasts, from the correspondent heterozygous clinically unaffected sibling, were obtained from Coriell Cell Repositories (Camden, NJ, USA) and were cultured in RPMI supplemented with 15% FBS.

### Antibodies

The following antibodies were used for Western Blot analysis: mAb anti-frataxin (MAB-10876, Immunological Sciences), mAb anti-tubulin (Sigma-Aldrich) and secondary antibody horseradish peroxidase-conjugated goat anti-mouse (Pierce).

### Chemicals

Proteasome inhibitors: MG132 (Sigma-Aldrich); DUB inhibitors: Ub-aldehyde (Biomol) and N-ethylmaleimide (NEM; Sigma-Aldrich). UCM were obtained from Enamine or were synthesized. The identity and purity of the compounds obtained were determined by TLC analysis and ^1^H-NMR.

### DNA constructs

The pIRES2–frataxin^1–210^ and pIRES2-frataxin^K147R^ constructs were previously described (Condò et al., 2006; Rufini et al., 2011). The HA-Ub construct was generated by M. Treier in Dirk Bohmann's lab (Treier et al., 1994).

### Immunoblotting

Total cell extracts were prepared in IP buffer (50 mM Tris–HCl, pH 7.5, 150 mM NaCl, 1% Nonidet P-40, 5 mM EDTA, 5 mM EGTA) supplemented with Complete protease inhibitor cocktail (Roche Diagnostics). For *in vivo* detection of ubiquitin conjugates, 10 μM MG132, 50 ng/ml Ub-aldehyde and 2 mM NEM were added to the lysis buffer. Protein extract (50 μg) was separated by 12% SDS–PAGE, blotted onto a nitrocellulose membrane and detected with specific antibodies. The immunoreactive bands were detected by ECL (GE Healthcare) and imaged with a ChemiDoc XRS system (Bio-Rad Laboratories). Densitometric analysis was performed using the ImageLab 4.1 Software (Bio-Rad Laboratories).

### Steady-state fluorescence measurements

Human recombinant frataxin precursor (aa 1-210) was expressed and purified by GenScript Corp., NJ, USA. Steady-state fluorescence spectra were recorded at 20 °C using an ISS PC1 fluorometer (Iss Inc, Champain, IL, USA). The affinity of frataxin for the ligands studied in this paper was determined by monitoring the decrease in Trp fluorescence upon the addition of these molecules to solutions. The concentration of the protein was 2 μM while the concentration of the ligands varied from 0.5 to 25 μM. At the excitation wavelength of 280 nm, emission spectra were recorded between 290 and 440 nm, using a 4 × 4 mm path-length quartz fluorescence microcuvette (Hellma GmbH & Co., Müllheim/Baden, Germany). The spectra were corrected using an instrument correction curve obtained with standard fluorescent compounds such as *N*-acetyl tryptophanamide (NATA). All measurements were also corrected for the inner-filter effect. To simulate the absorption of ligand molecules at the excitation and the emission wavelengths, two cuvettes (2 mm optical length), containing a solution of ligands in buffer, were placed along the excitation and emission pathways. The fluorescence of NATA was thus measured varying the concentration of quencher and four correction curves (one for each ligands) were obtained.

Data were then plotted as fractional loss of Trp fluorescence (Δ*F*/*F*0) *versus* ligand concentration. Experimental data were analyzed by nonlinear regression through a hyperbolic binding isotherm, using the Kaleidagraph program (Synergy Software).

### Enzyme assays

For determinations of aconitases and citrate synthase activity, FRDA lymphoblasts were washed twice with ice-cold Dulbecco's phosphate-buffered saline (DPBS) and lysed in CelLytic M buffer (Sigma-Aldrich) supplemented with Complete protease inhibitor cocktail EDTA-free (Roche) and 2 mM trisodium citrate. Citrate was included to prevent the inactivation of iron-sulfur cluster of aconitases. Total aconitase activity was measured spectrophotometrically at 340 nm using the BIOXYTECH Aconitase-340^TM^ Assay (OxisResearch^TM^ 21041). The assay reactions, containing 150 μg of cell extract, were performed following the supplier's procedure, with the exception of temperature incubation at 25 °C. Citrate synthase activity was assessed using 15 μg of cell extract with the Citrate Synthase Assay Kit (Sigma-Aldrich CS0720).

The aconitase activities were normalized with respect to citrate synthase ratios; 1 mU of enzyme was defined as the amount of protein that converted 1 nmol of NADP^+^ in 1 min at 25 °C.

### Frataxin modeling

The crystal structure of human frataxin (PDB code 1EKG) (Dhe-Paganon et al., 2000) was used as the reference structure. NMR solution structures (PDB code 1LY7, 15 models) (Musco et al., 2000) were aligned on the crystal structure using the program Combinatorial Extension (Shindyalov and Bourne, 1998). All the experimental structures were modeled with the software package Gromacs v. 4.6.5 to add missing atoms and to refine the models. Side chains flexibility was investigated with the program reduce. The refined crystal structure was used to generate 1000 protein models with tCONCOORD (Seeliger and de Groot, 2010).

### Frataxin binding site analysis

The experimentally determined models of frataxin were used to investigate the location of putative binding pockets on the solvent accessible surface of the protein using Metapocket (Huang, 2009), a freely available server that includes eight different methods: ConCavity (Capra et al., 2009), Fpocket (Le Guilloux et al., 2009), GHECOM (Kawabata, 2010), LIGSITE (Huang and Schroeder, 2006), PASS (Brady and Stouten, 2000), POCASA (Yu et al., 2010), Q-SiteFinder (Laurie and Jackson, 2005) and SURFNET (Laskowski, 1995). Mdpocket (Le Guilloux et al., 2009) was employed to investigate transient pockets using the computer-generated models obtained by tCONCOORD (Seeliger and de Groot, 2010).

### Molecular docking

AutoDock (Morris et al., 2009) and AutoDock/Vina (Trott and Olson, 2010) were used to conduct virtual screening experiments. Screening of large data sets was conducted with AutoDock/Vina using a rigid model for the protein. Focused libraries were modeled using AutoDock flexible approaches: flexible docking in which selected side chains are treated as flexible; conformational selections experiments conducted on the tCONCOORD-generated models (Seeliger and de Groot, 2010) were performed on selected ligands (UCM71 and UCM108).

### Cheminformatics and database searching

In-house database of commercially available compounds was screened to search for compounds containing the sulfonyl-hydrazone scaffold with aromatic substituents using the following criteria: MW < 500, logP < 5, no atoms with undefined stereo, no reactive groups (Oprea, 2000). More than 5000 compounds were identified and docked on the X-ray structure of frataxin. About 100 molecules were selected and bought for biological testing. Bioisosters of sulfonyl-hydrazones were modeled and screened with the docking procedures described above.

The following are the supplementary data related to this article.

Supplementary data to this article can be found online at http://dx.doi.org/10.1016/j.nbd.2014.12.011.

## Figures and Tables

**Table 1:** Chemical structure and activity of the compounds described in the present study.

Compound name	Structure	Activity
UCM53		+
UCM57		−
UCM71		+
UCM72		−
UCM108		+
